# Wheat stem rust recorded for the first time in decades in Ireland

**DOI:** 10.1111/ppa.13532

**Published:** 2022-02-09

**Authors:** Ayako Tsushima, Clare M. Lewis, Kerstin Flath, Stephen Kildea, Diane G. O. Saunders

**Affiliations:** ^1^ John Innes Centre Norwich Research Park Norwich UK; ^2^ Institute for Plant Protection in Field Crops and Grassland Julius‐Kuehn‐Institut (JKI) Kleinmachnow Germany; ^3^ Teagasc Oak Park, Carlow Ireland

**Keywords:** pathogenomics, plant pathology, *Puccinia graminis*, wheat stem rust

## Abstract

Wheat stem rust, caused by the fungus *Puccinia graminis* f. sp. *tritici* (Pgt), occurs in most wheat‐growing areas worldwide, and, in western Europe since 2013, has started to re‐emerge after many decades of absence. Following this trend across western Europe, in 2020, we also detected and recorded wheat stem rust for the first time in five decades in experimental plots across five locations in Ireland. To examine the potential origin of the Irish Pgt infection in 2020, we carried out transcriptome sequencing on 12 Pgt‐infected wheat samples collected across Ireland and compared these to 76 global *P*. *graminis* isolates. This analysis identified a close genetic relationship between the Irish Pgt isolates and those from Ethiopia collected in 2015 after a severe stem rust epidemic caused by the TKTTF Pgt race, and with the UK‐01 Pgt isolate that was previously assigned to the TKTTF race. Subsequent pathology‐based race profiling designated two Irish isolates and recent UK and French Pgt isolates to the TKTTF Pgt race group. This suggests that the Irish Pgt occurrence most probably originated from recent long‐distance windborne dispersal of Pgt urediniospores from neighbouring countries in Europe where we confirmed the Pgt TKTTF race continues to be prevalent. The identification of wheat stem rust in Ireland at multiple locations in 2020 illustrates that the disease can occur in Ireland and emphasizes the need to re‐initiate local monitoring for this re‐emergent threat to wheat production across western Europe.

## Graphical abstract

Wheat stem rust was recorded at multiple trial plots in Ireland in 2020 for the first time in five decades, with pathogen isolates typing to the TKTTF race that is common in western Europe.

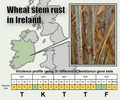

## 1 INTRODUCTION

Wheat stem rust, caused by the fungus *Puccinia graminis* f. sp. *tritici* (Pgt), has threatened wheat and barley production throughout history (Peterson, 2001). Today, it is a principal biotic constraint in most major wheat‐growing areas worldwide, with the potential emergence of new virulent strains that overcome varietal resistance a serious global concern (Gill et al., 2020). Such trepidation was substantiated by the appearance of the highly virulent Ug99 race (termed TTKSK) in Uganda in 1998, which dramatically changed the scale of epidemics, consigning 80% of the world's wheat varieties vulnerable to this one race (Singh et al., 2011). Since then, a number of additional races of concern have emerged and spread widely. For instance, the TKTTF race that is thought to have originated in the Middle East, spread into Ethiopia and caused severe epidemics in 2013 and 2014 (Olivera et al., 2015). In parallel, the TKTTF race and its variants were identified in 2013 in sporadic outbreaks in several European countries, including Germany (Firpo et al., 2017), Denmark and the UK (Lewis et al., 2018). This signified a significant turning point for western Europe that had remained largely free of wheat stem rust since the mid‐twentieth century. The long period of absence of wheat stem rust in western Europe has also moved attention away from resistance breeding and inevitably led to little resistance being present in modern European germplasm; in the UK it was shown that more than 80% of commonly cultivated wheat varieties tested were highly susceptible to an isolate of the TKTTF race (Lewis et al., 2018). This lack of stem rust resistance in western Europe now poses a serious concern regarding the potential for future widespread epidemics and emphasizes the critical need for continuous monitoring (Saunders et al., 2019).

Over the past decade more significant outbreaks have been reported in Europe. In 2016, a large wheat stem rust outbreak occurred in Sicily, and Sweden reported a new outbreak on late‐maturing wheat and barley in 2017 (Berlin, 2017; Bhattacharya, 2017). Wheat stem rust epidemics are the result of rapid proliferation of the asexual stage of the Pgt lifecycle with vast quantities of wind‐dispersed urediniospores transmitting disease between susceptible gramineous host plants (Roelfs et al., 1992). With the cool winter climate in north‐west Europe precluding overwintering of Pgt urediniospores, they are created anew each year or transmitted long‐distance on wind currents from warmer climates in the south (Hogg et al., 1969). Hence, inbound Pgt urediniospores frequently reach northern Europe wheat fields too late to cause significant damage (Van Der Plank, 1963). However, healthy wheat crops can be damaged by stem rust infection within just 3 weeks of harvest, by rapid disease development that interrupts nutrient flow and leads to severely wrinkled and nutritionally inferior grain (Roelfs et al., 1992). Therefore, even very late season exotic incursions of Pgt urediniospores can severely impact yield if sufficiently abundant and arriving under conditions conducive to supporting disease development.

Within western Europe, in‐country inoculum is generated much earlier in the growing season through infection of the pathogen's alternate hosts, which include *Berberis* and *Mahonia* spp. (Roelfs et al., 1992). In the spring, overwintering hardy teliospores germinate to form basidiospores that can infect susceptible *Berberis* species. Following sexual recombination, aeciospores then develop within cluster cups (“aecia”) that form on the underside of *Berberis* leaves, that, once liberated in the spring, are transmitted on air currents to infect neighbouring gramineous hosts (Roelfs et al., 1992). Due to the critical role of *Berberis* in the Pgt lifecycle, sanitation efforts were initiated to enforce removal of the most susceptible species known as common barberry (*Berberis vulgaris*) in Europe and North America, with the first laws passed as early as 1660 in Rouen, France (McKay, 1957). Removal of the fungus's alternate host intensified throughout the nineteenth century and was extremely successful in controlling stem rust epidemics in north‐western Europe. However, where barberry bushes remain, small sporadic outbreaks can still occur and have increased in prevalence in recent years as legislation restricting *Berberis* planting has lapsed, and this popular hedgerow shrub has become a more frequent feature in the landscape (Barnes et al., 2020).

Historically, in Ireland, stem rust was known to occur in the southern half of the country and classified as endemic in districts where common barberry (*B*. *vulgaris*) was prevalent (McKay, 1957). Accordingly, the last severe stem rust epidemics occurred in 1955 and 1963 and were thought to be indigenous in origin. For instance, in 1955, favourable climatic conditions led to rapid spread of the disease across southern Irish counties beyond the immediate vicinity of barberry bushes (Hogg et al., 1969; McKay, 1957) with yield losses in experimental wheat plots as high as 33% (Hogg et al., 1969). This swiftly led to the addition of *B*. *vulgaris* to the Noxious Weed Act in 1958 to inhibit its growth and spread across Ireland (Anonymous, 1936). However, small amounts of *B*. *vulgaris* are still present in Ireland, after many decades of stem rust absence (Anonymous, 2021b). Following the trend across western Europe in the past decade, in 2020, we also detected and recorded wheat stem rust for the first time in five decades in experimental plots across multiple locations in Ireland. We used comparative genomic analysis and pathology‐based race profiling to identify the Pgt race concerned and investigated the presence of *Berberis* hedgerows in the local vicinity of field infections. The results were used to conclude the most likely origin of the Pgt race identified.

## 2 MATERIALS AND METHODS

### 2.1 Visual identification of Irish stem rust lesions and *P*. *graminis* urediniospores

To provide an initial confirmation of Irish stem rust infections, stems displaying typical lesions were inspected and photographed using a Leica E24HD dissecting microscope. Uredinia were scraped with a fine needle and urediniospores suspended in water on a microscope slide and examined and photographed at 20× magnification using a Leica DM1000 microscope with a Leica ICC50W camera and associated Leica Application Suite v. 4.7.1.

### 2.2 Collection of *P*. *graminis*‐infected plant samples

A total of 24 *P*. *graminis*‐infected bread wheat (*Triticum aestivum*), durum wheat (*T*. *durum*) or barley (*Hordeum vulgare*) stem or leaf samples were collected in Ireland, France, Italy and the UK between 2018 to 2020 and stored in RNAlater solution (Thermo Fisher Scientific) to maintain nucleic acid integrity (Table S1). In addition, we collected three wheat samples from plants subjected to controlled infections with spores derived from a single rust aecium identified on a *B*. *vulgaris* leaf collected around Kelso (UK), described below.

### 2.3 Collection of wheat samples infected with spores derived from a *P*. *graminis* aecium

First, to confirm that the aecium collected in Kelso was *P*. *graminis*, DNA was extracted from a portion of the aecium using the DNeasy Plant Mini Kit (QIAGEN) and the internal transcribed spacer (ITS) region amplified using primers 5ITS (5′‐GGAAGTAAAAGTCGTAACAAGGT‐3′) and 3ITS (5′‐ACTCCTTGGTCCGTGTTTCA‐3′) (Orton et al., 2019). The resulting PCR product was purified using the Qiaquick PCR purification kit (QIAGEN) and sequenced (Genewiz). The amplicon sequence was then used in a BLAST search against the NCBI non‐redundant database (NCBI‐Resource‐Coordinators, 2018).

The remaining portion of the *P*. *graminis* aecium was stored initially in damp conditions to induce release of aeciospores for up to 3 h before being applied with gentle rubbing to the leaves of the wheat variety Vuka at seedling stage. After infection, seedlings were kept in the dark at 16°C and high relative humidity for 24 h and then plants were moved to a controlled environment room under long‐day conditions (16 h light/8 h dark) and 18/12°C cycle. Once pustules were evident on the leaf surface, a single leaf segment was cut that contained an isolated single pustule and stored in RNAlater solution. Two further single isolated pustules were cut, suspended in Novec 7100 engineered fluid (Sigma‐Aldrich) and used independently for spray inoculation of 3‐week‐old Vuka plants following procedures described previously (Sørensen et al., 2016). Plants were incubated as described above and once pustules formed, a single *P*. *graminis*‐infected leaf sample was collected from each infection and stored in RNAlater solution.

### 2.4 RNA‐seq of *P*. *graminis*‐infected plant samples

All 27 *P*. *graminis*‐infected leaf and stem samples were subjected to RNA extraction using an RNeasy Plant Mini Kit (QIAGEN) and the quality and quantity of extracted RNA assessed using an Agilent 2100 Bioanalyzer. Genewiz constructed complementary DNA libraries using the TruSeq RNA Sample Preparation Kit (Illumina) and sequenced these libraries on an Illumina NovaSeq machine, generating 150 bp paired‐end reads. Adapter and barcode trimming, and quality filtering were performed on the paired‐end reads using FASTX‐Toolkit v. 0.0.13.2.

### 2.5 Genome sequencing of *P*. *graminis* isolates

Dried urediniospores from four *P*. *graminis* f. sp. *secalis* (Pgs) and three Pgt isolates collected in Germany (Table S1) were independently subjected to DNA extraction using the cetyltrimethylammonium bromide (CTAB) method with minor modification (Chen et al., 1993). In brief, a total of 80–100 mg of urediniospores were mixed with sterile sand (50–70 mesh particle size; Sigma‐Aldrich) and ground to a fine powder with a pestle and mortar. To each sample a total of 2 ml of prewarmed CTAB extraction buffer (50°C; 137 mM d‐sorbitol, 34 mM *N*‐lauroylsarcosine sodium salt, 24 mM centrimide, 0.8 M NaCl, 22 mM EDTA, 10 g/L polyvinylpolypyrrolidone) and 5 µl proteinase K (20 mg/ml; Thermo Fisher Scientific) was added, followed by incubation at 50°C for 2 h. One volume of chloroform:isoamyl alcohol (24:1; Thermo Fisher Scientific) was added to each sample prior to centrifugation at 1922 × *g* for 10 min. The aqueous phase was transferred to a clean tube, 20 µl RNase (10 mg/ml; Thermo Fisher Scientific) was added, and samples were incubated at room temperature for 1 h. Following a second round of chloroform:isoamyl alcohol addition, and centrifugation, the aqueous phase was transferred to a clean tube and 1 volume of isopropanol added. Samples were incubated at −20°C overnight, then centrifuged at 1922 × *g* for 10 min. The supernatant was removed, and the resulting pellet rinsed twice in 70% ethanol and then air dried for 30 min before resuspension in 30 µl TE buffer (10 mM Tris‐HCl, 1 mM EDTA, pH 8.0). The quantity and quality of DNA was assessed using the Qubit 3.0 (Qubit). Genewiz prepared gDNA libraries using the TruSeq DNA Library Preparation Kit (Illumina) and the libraries were subsequently sequenced on an Illumina NovaSeq machine generating 150 bp paired‐end reads.

### 2.6 Alignment of reads to the Pgt reference genome

In addition to the 34 *P*. *graminis* data sets generated, we sourced publicly available RNA‐seq and genomic data for an additional 54 *P*. *graminis* isolates for inclusion in our analyses (Table S1). All RNA‐seq and genomic DNA reads from a total of 88 samples were independently aligned to the Pgt reference genome (isolate 21–0, haplotype B; Li et al., 2019), using STAR v. 2.5a for RNA‐seq data sets (Dobin et al., 2013) and BWA v. 0.75 for genomic DNA data sets (Li & Durbin, 2009). RNA‐seq alignments were reformatted using SplitNCigarReads from GATK v. 4.0.0.0 to span introns (McKenna et al., 2010). Single‐nucleotide polymorphisms (SNPs) were identified using SAMtools v. 0.1.19 (Li et al., 2009), with a minimum depth of coverage of 20× for RNA‐seq data and 10× for all genomic data as described previously (Hubbard et al., 2015). At each SNP site, allelic frequencies were assessed and those ranging from 0.2 to 0.8 were categorized as heterokaryotic sites and those with other frequencies as homokaryotic sites. Read frequencies for heterokaryotic SNP sites were analysed for the 27 RNA‐seq data sets generated from Pgt‐infected leaf and stem material and plotted using ggplot2 in R (Villanueva & Chen, 2019). SNP sites that induced synonymous and nonsynonymous substitutions were identified using SnpEff v. 3.3a (Cingolani et al., 2012).

### 2.7 Phylogenetic analysis of *P*. *graminis* isolates

Phylogenetic analyses of the 88 *P*. *graminis* isolates were performed considering only gene coding regions to avoid over‐representation of Pgt isolates subjected to whole‐genome sequencing compared to those subjected to transcriptome sequencing, as described previously (Lewis et al., 2018). The third codon position of 7812 gene models (3,869,584) with ≥80% breadth of coverage for all *P*. *graminis* isolates was used to generate a maximum‐likelihood tree with RaxML v. 8.0.20 using the rapid bootstrap algorithm and 100 replicates (Stamatakis, 2014). Two *P*. *graminis* f. sp. *avenae* (Pga) isolates were included as an outgroup in the phylogeny and phylogenetic trees were visualized using iTOL v. 6.1.2 (Letunic & Bork, 2021).

### 2.8 Population genetic analysis

Population subdivisions among the 86 *P*. *graminis* isolates (excluding the two Pga isolates) were investigated using discriminant analyses of principal components (DAPC) implemented in the adegenet v. 2.1.1 package in the R environment (Jombart, 2008). A total of 16,639 biallelic SNP sites that introduced a synonymous change in at least one isolate were used for the analyses. The optimum number of clusters was determined using the Bayesian information criterion and subsequent DAPC used to assign individuals to distinct population clusters.

### 2.9 Analyses of variation in fungicide target genes

Major fungicide classes that are used to control wheat rust include succinate dehydrogenase inhibitors (SDHIs) that target the SDH complex, encoded by the *SdhA* to *SdhD* genes and demethylation inhibitors (DMIs) that inhibit the 14α‐demethylase enzyme encoded for by one or more *Cyp51* gene(s) (Cook et al., 2021). To assess the 86 *P*. *graminis* isolates (excluding the two Pga isolates) for sequence variation in these genes, we first identified orthologs of *SdhA* to *SdhD* and *Cyp51* by conducting BLASTP searches of the Pgt proteome (isolate 21‐0; Li et al., 2019) using protein sequences for SdhB to SdhD and Cyp51 from *Puccinia striiformis* f. sp. *tritici* (Cook et al., 2021) and the SdhA protein sequence from *Saacchromyces cerevisiae* identified in NCBI as queries (Table S2). To analyse sequence variation between *P*. *graminis* isolates, all heterokaryotic and homokaryotic SNPs determined from each individual alignment were incorporated into synthetic sequences for the five fungicide target genes for each *P*. *graminis* isolate. The five genes were evaluated for synonymous and nonsynonymous mutations for each *P*. *graminis* isolate through comparisons against the Pgt reference gene set (isolate 21‐0, haplotype B; Li et al., 2019) using Geneious Prime v. 2020.2.2 (Biomatters Ltd).

### 2.10 Virulence phenotyping of *P*. *graminis* isolates

Two Irish (IE‐01 and IE‐12), one French (FR‐11) and one UK (UK‐02) Pgt isolates were selected for virulence phenotyping and their urediniospores were multiplied by successive rounds of inoculation on the susceptible wheat variety Vuka. For each round of inoculation, 7‐day‐old Vuka seedlings were treated with maleic hydrazide (50 ml per pot of 0.2 g/L water solution) to retard seedling growth, then seedlings were inoculated with a suspension of urediniospores in Novec 7100 engineered fluid (Sigma‐Aldrich) following procedures described previously (Sørensen et al., 2016). Approximately 20 mg of purified urediniospores for each of the four Pgt isolates was then used to inoculate a standard wheat differential set of 20 wheat varieties, which include the host resistance genes *Sr5*, *Sr21*, *Sr9e*, *Sr7b* (set 1); *Sr11*, *Sr6*, *Sr8a*, *Sr9g* (set 2); *Sr36*, *Sr9b*, *Sr30*, *Sr17* (set 3); *Sr9a*, *Sr9d*, *Sr10*, *SrTmp* (set 4); and *Sr24*, *Sr31*, *Sr38*, and *SrMcN* (set 5). For each wheat line, up to 10 plants were tested under controlled environment conditions under long‐day conditions (16 h light/8 h dark) and 18/12°C cycle. However, due to the temperature sensitivity of the *Sr21* gene (Chen et al., 2018), Pgt inoculations on the line containing that gene were instead incubated under long‐day conditions (16 h light/8 h dark) and a 16/16°C cycle, with the previously characterized Pgt UK‐01 isolate used as a control (Lewis et al., 2018). At 21 days postinoculation Pgt infection types were assessed on the first seedling leaf, following the scoring system established by Stakman et al. (1962), where 0, ; (fleck), 1−, 1, 1+, 2−, 2 and 2+ were considered as representing low compatibility and 3, 3+ and 4 represented high compatibility.

## 3 RESULTS

### 3.1 Wheat stem rust identified in multiple locations in Ireland in 2020

**FIGURE 1:**
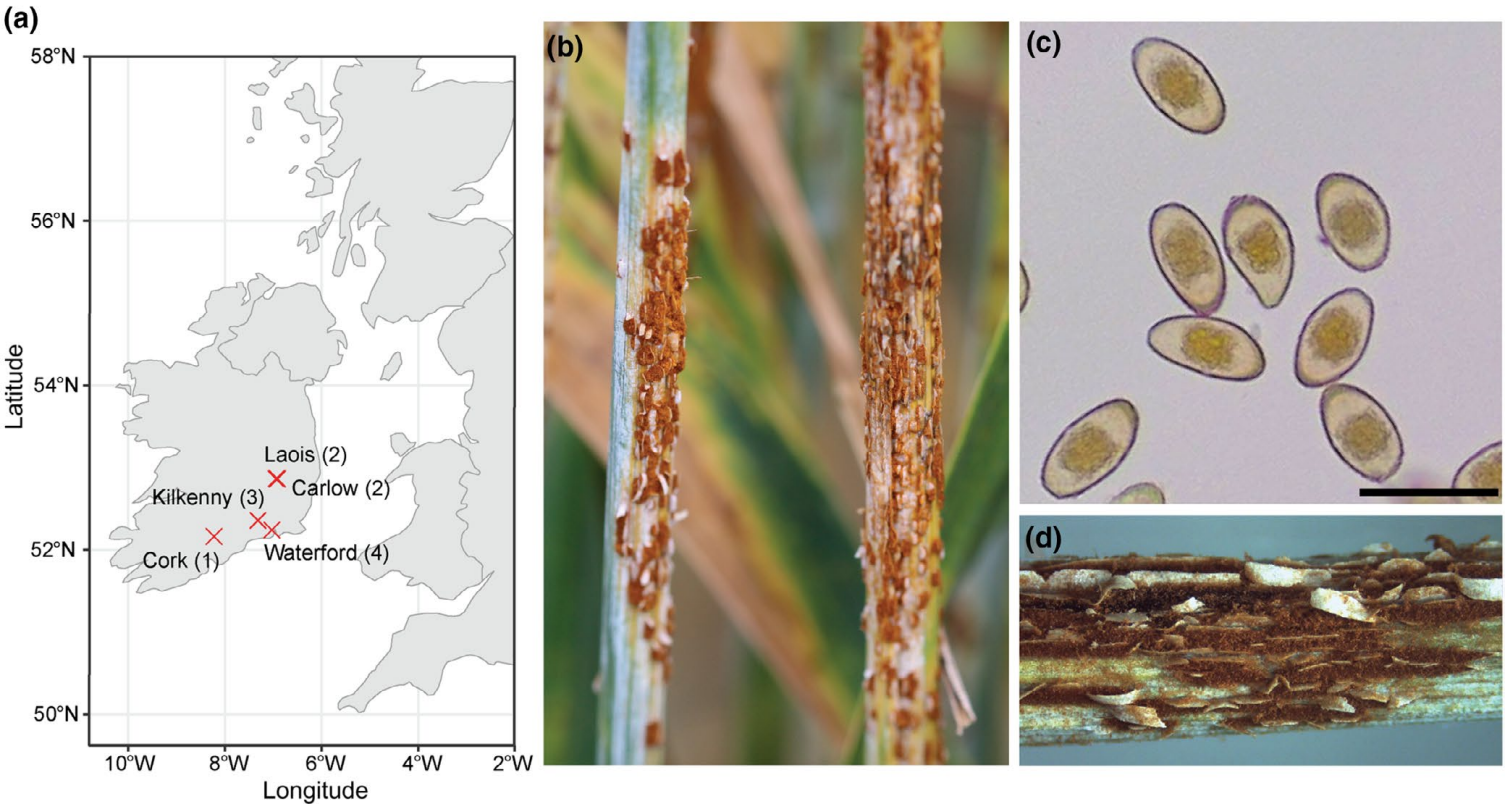
Wheat stem rust was identified at five locations in southern Ireland in July 2020. (a) Wheat stem rust symptoms were identified across a genetically diverse array of winter wheat varieties, breeding and inbred lines in agrochemical field trial sites in Laois, Carlow, Kilkenny, Cork and Waterford (red crosses). Numbers represent the number of samples taken and subjected to transcriptome sequencing from each location. (b) Representative image of orange uredinia, typical of stem rust infection, that were found on the stems of wheat plants at each location. (c) Micrographs of urediniospores recovered from infected wheat plants. Scale bar represents 50 µm. (d) Image of erumpent black telia full of teliospores that were also visible on certain wheat stems

In July 2020, we recorded wheat stem rust (Pgt) for the first time in five decades in Ireland in untreated bread wheat (*T*. *aestivum*) trial plots at five locations: Laois, Carlow, Kilkenny, Cork and Waterford (Figure 1a). In each case, multiple plants of each wheat variety exhibited stem lesions filled with brick‐red spiny oval urediniospores, typical of stem rust infection (Figure 1b,c), with erumpent black telia full of teliospores visible on certain wheat stems (Figure 1d). Infections were identified on a genetically diverse array of winter wheat varieties, breeding and inbred lines (Table S1). To establish the likelihood of the infections arising from in‐country inoculum originating on *Berberis* in the local vicinity or from an exotic incursion of Pgt urediniospores, we conducted a brief evaluation of the local area for *Berberis* plantings and consulted publicly available *Berberis* location records (Anonymous, 2021b; Lockton & Hughes, 2021). Our analyses failed to identify any *Berberis* hedgerows in close proximity to any of the five locations. This suggested that the stem rust infections occurring in 2020 in Ireland were more likely to have been derived from an influx of windborne Pgt urediniospores, either from a distal location in Ireland or a neighbouring country.

### 3.2 Irish Pgt isolates are genetically similar to Pgt isolate UK‐01

**FIGURE 2:**
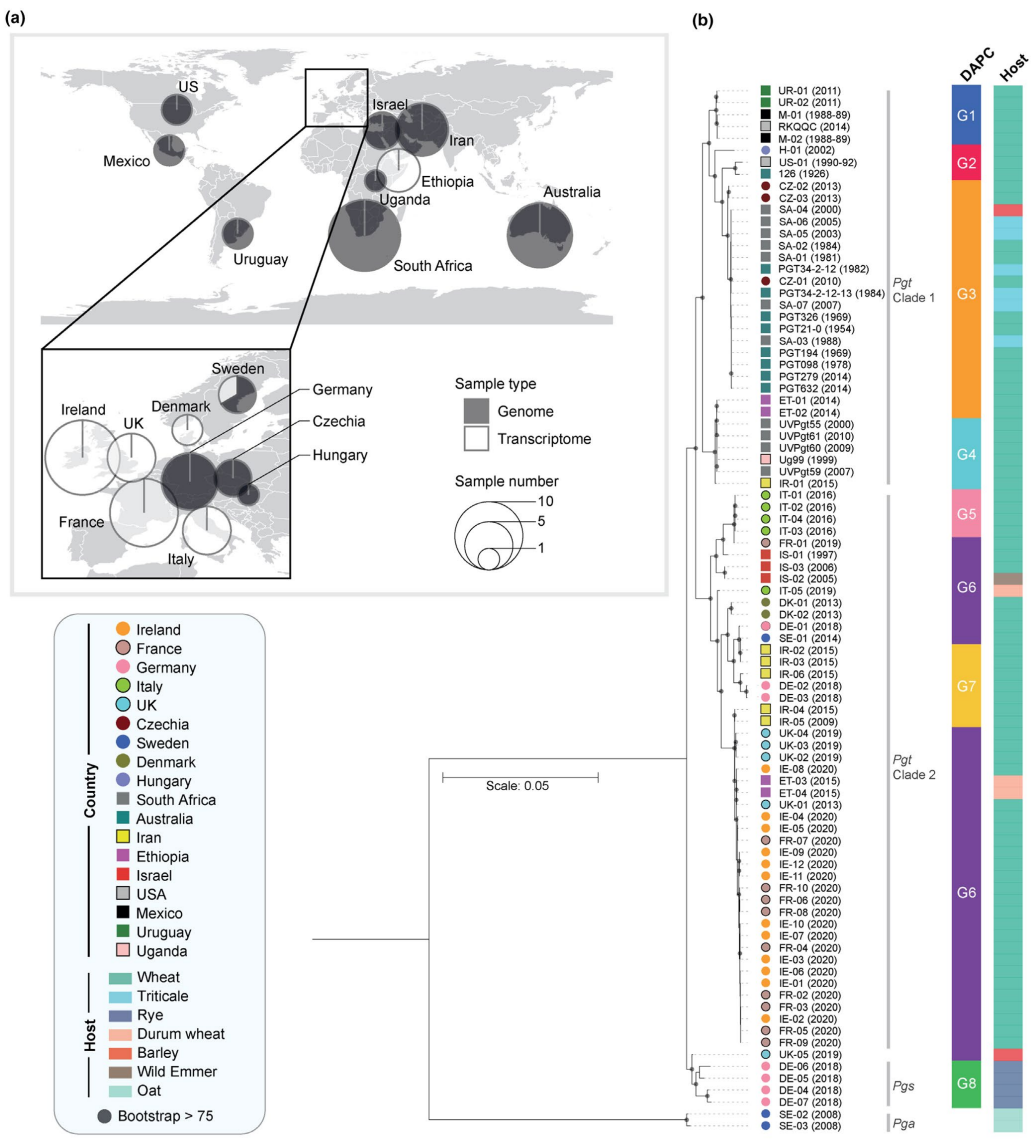
The Irish *Puccinia graminis* f. sp. *tritici* (Pgt) isolates sampled in 2020 are genetically closely related to those belonging to the TKTTF race group. (a) A total of 88 genomic and transcriptomic data sets were sourced for *P*. *graminis* isolates from 18 countries, with an enrichment of representation of *P*. *graminis* isolates from European countries (48 *P*. *graminis* isolates). (b) Phylogenetic analysis and population genetic clustering illustrated that the Irish Pgt isolates collected in 2020 were genetically similar to those belonging to the TKTTF race. Phylogenetic analysis was performed using the third codon position of 7812 gene models (3,869,584 sites) and a maximum‐likelihood approach with two *P*. *graminis* f. sp. *avenae* (Pga) isolates used as outliers, with the two major Pgt clades indicated (Clade 1 and Clade 2). Multivariate analysis with discriminant analysis of principle components (DAPC) was performed with 16,639 biallelic synonymous single nucleotide polymorphism (SNP) sites and grouped the Pgt and *P*. *graminis* f. sp. *secalis* (Pgs) isolates into eight distinct population clusters (G1–G8). In the DAPC bar charts, each bar represents the estimated membership fractions for each individual to each of the eight population clusters. Population clusters (G1–G8) are represented by distinct colouring. For each isolate, their country and host of origin are shown

To examine the potential origin of the Irish wheat stem rust infection in 2020, we sought to generate, and gather publicly available genomic and transcriptomic data for comparative population analyses (Figure 2a). A total of 12 Pgt‐infected wheat stem samples were collected across the five experimental plots in Ireland and, to enrich the representation of Pgt isolates from European countries in subsequent analyses, 11 additional Pgt‐infected leaf and stem samples were collected in France in 2019 and 2020 (10 samples) and Italy in 2019 (one sample). We also collected four additional UK samples that included a sample from a single barley plant that displayed typical stem rust symptoms in summer 2019 (UK‐05) (Orton et al., 2019), and three wheat samples (UK‐02, UK‐03 and UK‐04) from plants subjected to controlled infections with spores derived from a single rust aecium identified on *B*. *vulgaris*. All aforementioned samples were subjected to transcriptome sequencing. We also conducted genome sequencing on four purified Pgs isolates and sourced publicly available genomic and transcriptomic data from 52 additional Pgt isolates and two Pga isolates.

High‐quality reads for all 88 genomic and transcriptomic *P*. *graminis* data sets were aligned to the Pgt reference genome (isolate 21‐0; Li et al., 2019; Table S3). To ensure the 27 transcriptomic data sets we generated from Pgt‐infected leaf and stem samples represented a single Pgt genotype, read frequencies were assessed at heterokaryotic SNP sites. The majority of samples displayed a single mode at 0.5, typical of a dikaryotic distribution (Hubbard et al., 2015; Figure S1). One sample (UK‐05) displayed a wider ranging distribution, although the presence of only two alleles in this case would still tend to support a single genotype. To investigate the genetic similarity between the *P*. *graminis* isolates, we conducted phylogenetic analyses (3,869,584 sites) and population genetic analyses using DAPC (16,639 synonymous SNP sites). Analysis of the Bayesian information criterion in the DAPC indicated eight as the optimum clustering solution (Figure 2b; Figures S2 and S3). In the phylogeny, the Pgs and Pga isolates formed their own distinct clades, with the Pgs clade branching at a position immediately basal to all Pgt isolates and Pga isolates forming a clear outgroup. The DAPC also assigned all Pgs isolates to a separate population cluster, G8. We noted that the UK *P*. *graminis* sample from barley taken in 2019 (UK‐05) grouped in the phylogeny with the four Pgs isolates (DE‐04 to 07), yet in DAPC it was assigned to population cluster G6 that contained Pgt isolates (Figure 2b). The inability for genetic analyses to clearly assign UK‐05 to Pgs or Pgt probably reflects the documented close genetic proximity between the two formae speciales (Berlin et al., 2013).

In the phylogeny, Pgt isolates were subdivided between two major clades, which was also reflected in the DAPC with population clusters G1 to G4 representing Pgt isolates in clade 1 and clusters G5 to G7 representing Pgt isolates in clade 2. With the exception of one Pgt isolate from Hungary and three from the Czech Republic, all European Pgt isolates were clustered together in clade 2, with the Irish, French and UK Pgt isolates forming a single monophyletic group with high support (Figure 2b). The Irish, French and UK Pgt isolates were also grouped closely with Pgt isolates from Ethiopia collected in 2015, after a severe stem rust epidemic caused by the TKTTF Pgt race (Olivera et al., 2015), and the UK‐01 Pgt isolate that was previously confirmed as belonging to the TKTTF race (Lewis et al., 2018). The close genetic proximity between the Irish, French, UK and Ethiopian TKTTF isolates was further supported by DAPC that grouped them into a single population cluster (G6), which also contained recent Pgt isolates collected in Israel, Italy, Denmark, Germany and Sweden. This suggests that the Irish Pgt isolates identified in 2020 may also belong to the TKTTF race.

### 3.3 The Pgt TKTTF race confirmed in Ireland, France and the UK

**FIGURE 3:**
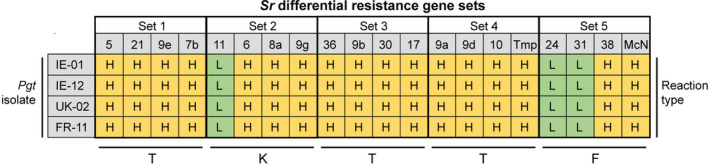
Virulence phenotyping of Irish, French and UK *Puccinia graminis* f. sp. *tritici* (Pgt) isolates supports assignment to the TKTTF race group. Two Irish (IE‐1 and IE‐12), one French (FR‐11) and one UK (UK‐02) Pgt isolates were inoculated onto a series of standard differential wheat line sets that contain 20 monogenic stem rust (*Sr*) resistance genes (Stakman et al., 1962). Disease severity on each line was assessed in seedling assays approximately 21 days after inoculation, with all four Pgt isolates classed as virulent (high reaction type; H) on wheat lines with the resistance genes *Sr5*, *Sr21*, *Sr9e*, *Sr7b*, *Sr6*, *Sr8a*, *Sr9g*, *Sr36*, *Sr9b*, *Sr30*, *Sr17*, *Sr9a*, *Sr9d*, *Sr10*, *SrTmp*, *Sr38* and *SrMcN*, and avirulent (low reaction type; L) on wheat lines harbouring *Sr11*, *Sr24* and *Sr31*. Following the North American Pgt nomenclature system (Roelfs & Martens, 1988) the reaction types were used to assign all four Pgt isolates to the TKTTF race group

To examine the virulence profiles and unequivocally assign the recent Irish, French and UK Pgt isolates to specific race group(s), we inoculated representative isolates from each location onto a series of standard differential wheat line sets that contain 20 monogenic stem rust (*Sr*) resistance genes (Stakman et al., 1962). Disease severity on each line was assessed in seedling assays approximately 21 days after inoculation, with all four Pgt isolates selected giving similar results; all Pgt isolates were classed as virulent (high reaction type) on wheat lines with the resistance genes *Sr5*, *Sr21*, *Sr9e*, *Sr7b*, *Sr6*, *Sr8a*, *Sr9g*, *Sr36*, *Sr9b*, *Sr30*, *Sr17*, *Sr9a*, *Sr9d*, *Sr10*, *SrTmp*, *Sr38* and *SrMcN* and classed as avirulent (low reaction type) on wheat lines harbouring *Sr11*, *Sr24* and *Sr31* (Figure S4). Following the North American Pgt nomenclature system (Roelfs & Martens, 1988), we used the reaction types across these 20 wheat lines to assign all four Pgt isolates to the TKTTF race group (Figure 3). As this race is also present in continental Europe and the UK, our analyses support a recent incursion of the TKTTF Pgt race into Ireland and confirm the continued presence of this race in neighbouring UK and France where it has been documented since 2013 (Hovmøller, 2021; Lewis et al., 2018).

### 3.4 No evidence of mutations linked to a decrease in SDHI or DMI sensitivity for Pgt

During the recent stem rust occurrence in Ireland, we noted that infections were only identified in untreated control plots in agrochemical field trials, suggesting that chemical application may have limited further spread. This led us to also investigate sequence variation in the fungicide target genes *SdhA*, *SdhB*, *SdhC*, *SdhD* and *Cyp51* for Pgt. The homologs of each of the aforementioned genes were identified in the Pgt reference isolate (isolate 21‐0; Li et al., 2019) through BLAST searches. Sequence alignments for each of the five fungicide target genes were analysed for the presence specifically of nonsynonymous substitutions. A total of six variable and nonsynonymous nucleotide positions were identified in *SdhA*, five in *SdhB*, eight in *SdhC*, 11 in *SdhD* and 13 in *Cyp51* across the 86 global *P*. *graminis* isolates analysed (Table 1). None of the nonsynonymous mutations in any of the four genes were at positions analogous to those previously linked to a decrease in fungicide sensitivity for other fungal species (Cook et al., 2021).

**TABLE 1:** Variation in succinate dehydrogenase inhibitor (SDHI) and demethylation inhibitor (DMI) fungicide target genes identified in the 86 global *Puccinia graminis* isolates analysed

Gene	CDS position	CDS change	Amino acid position	Amino acid change	No. of isolates with homokaryotic variant	No. of isolates with heterokaryotic variant
*SdhA*	61–62	GC→AG	21	A→S	1	5
	62	C→G	21	A→G	0	1
	118	G→A	40	V→I	0	1
	812	C→T	271	T→I	0	3
	1428	C→A	476	N→K	0	1
	1459	T→C	487	S→P	0	35
*SdhB*	59	T→C	20	V→A	0	10
	68	C→T	23	S→L	1	14
	127	G→C	43	E→Q	0	8
	134	A→G	45	H→R	0	1
	464	A→G	155	D→G	0	1
*SdhC*	20	A→G	7	Q→R	0	5
	35	G→C	12	R→P	0	2
	73	A→C	25	T→P	0	2
	74	C→A	25	T→N	0	29
	99	G→C	33	L→F	0	30
	103	A→G	35	S→G	0	3
	108	G→T	36	L→F	0	2
	415	A→G	139	I→V	0	1
*SdhD*	14	C→G	5	T→R	4	18
	65	G→A	22	S→N	4	5
	92	C→A	31	T→K	40	27
	97	C→T	33	H→Y	0	1
	193	G→T	65	A→S	0	9
	275	A→G	92	Y→C	0	2
	308	C→A	103	S→Y	7	27
	310–311	CC→AA	104	P→K	7	27
	319	A→C	107	T→P	1	4
	383	A→G	128	K→R	0	5
	541	G→A	181	V→I	0	29
*Cyp51*	52	T→G	18	F→V	0	7
	145	A→G	49	T→A	1	7
	178	A→G	60	I→V	0	1
	235	A→G	79	K→E	0	5
	316	A→G	106	I→V	0	1
	388	A→G	130	T→A	1	1
	779	A→G	260	N→S	30	14
	1291	C→G	431	H→D	2	8
	1528	A→G	510	N→D	47	29
	1543	C→G	515	P→A	0	5
	1573	A→C	525	I→L	38	29
	1588	G→A	530	V→I	0	2
	1610	C→T	537	T→I	37	20

## 4 DISCUSSION

Increasing reports of wheat stem rust incidences across western Europe since 2013 have raised concern that this disease could once again re‐establish in this region (Saunders et al., 2019). However, to date disease incidences have been largely constrained to regional or sporadic infections (Hovmøller, 2021). This was also the case during the Irish outbreak in 2020 that appeared confined to the south of the country and occurred on a small number of plants in just five identified locations. In the present study, we also confirmed that all Pgt samples taken during the Irish outbreak were genetically closely related to each other and to Pgt samples from neighbouring countries of France and the UK that were collected in the same time period. The Irish, French and UK Pgt isolates also shared genetic similarity to the UK‐01 Pgt isolate that was previously typed to the TKTTF Pgt race (Lewis et al., 2018). Accordingly, pathology tests of two Irish, one French and one more recent UK Pgt isolate confirmed that they belong to the TKTTF Pgt race, which is currently one of the most common physiological races recorded in north‐western Europe (Firpo et al., 2017; Hovmøller, 2021; Lewis et al., 2018). This suggests that the stem rust outbreak in Ireland in 2020 was probably derived from wind‐dispersed urediniospores recently arriving from continental Europe and/or the UK where the TKTTF Pgt race is currently located.

In Ireland, the above average rainfall in June to August 2020 (Anonymous, 2020) appears to have created the ideal humid and warm conditions needed for Pgt urediniospore germination and disease development. Typically, the low winter temperatures prevent Pgt urediniospores from persisting between growing seasons in north‐western Europe with disease created either by windborne urediniospores entering from warmer regions or neighbouring *Berberis* hedgerows acting as a local source of inoculum. However, the 2019/2020 winter was exceptionally warm, with Europe experiencing the warmest winter on record, driven by a positive North Atlantic Oscillation (Hardiman et al., 2020). Hence, it is possible that urediniospores entering Ireland in the preceding season could have persisted until conditions became conducive to support Pgt germination and disease establishment. However, regardless of the timing of entry, the development of wheat stem rust appeared to occur too late in the growing season to initiate the scale of disease required to have an appreciable impact on wheat yields. Disease symptoms were also confined to control plots within agrochemical trial sites that were untreated with fungicide. This indicates that fungicide application in adjacent plots was very effective in controlling disease development.

The control of wheat stem rust is primarily reliant on a combination of routine fungicide application and the use of wheat varieties resistant to infection. However, little genetic resistance is present in current European germplasm at seedling or adult plant stage (Flath et al., 2018; Saunders et al., 2019), placing increased reliance on chemical control in this region. To date, although fungicides vary in their efficacy to control wheat stem rust (Wanyera et al., 2009), no reports of widespread field failure have been recorded. However, fungicide resistance‐associated mutations have recently been reported in the related wheat yellow rust pathogen (*P*. *striiformis* f. sp. *tritici*) (Cook et al., 2021) and the Asian soybean rust pathogen, *Phakopsora pachyrhizi* (Simoes et al., 2018). This indicates that cereal rusts are not immune to the evolution of fungicide resistance (Cook et al., 2021) and highlights the need for re‐initiation of resistance breeding in western Europe to heighten our defences in case wheat stem rust becomes re‐established in this region.

In Ireland, previous wheat stem rust outbreaks in the 1950s and 1960s were thought to be indigenous in origin and caused by Pgt inoculum originating on *Berberis* hedgerows neighbouring cereal fields (Hogg et al., 1969). Removal of the Pgt alternate host (*Berberis* spp.) can be exceptionally beneficial in reducing early season inoculum in regions where the pathogen is unable to overwinter in its urediniospore state and to curtail the spawning of new races through sexual recombination (Peterson, 2001). Despite the addition of *B*. *vulgaris* to the Noxious Weed Act in 1958, this highly susceptible species is still found in Ireland and, as suggested by the Agricultural Instructors in 1955, is probably more widely distributed than recognised (McKay, 1957). In 2020, a significant shift from winter to spring wheat and barley varieties occurred due to adverse weather conditions in autumn 2019 limiting winter planting; for instance, the number of hectares dedicated to winter wheat declined by 41.4% and spring wheat increased by 200.5% (Anonymous, 2021a). Future shifts towards later maturing spring wheat and barley varieties could diminish the protection afforded by the earlier maturing winter varieties that have been effective in western Europe at preventing inoculum build‐up (Zhao et al., 2016). As wheat crops can be significantly damaged by stem rust infection within just 3 weeks of harvest (Roelfs et al., 1992), the maturation delay afforded by spring varieties could provide the necessary window for stem rust to take hold and impact yields.

The identification in 2020 of wheat stem rust in Ireland for the first time in five decades at multiple locations demonstrates that wheat stem rust can occur in Ireland and emphasizes the need to remain vigilant. The lack of genetic resistance against wheat stem rust in European germplasm (Lewis et al., 2018; Saunders et al., 2019) and evidence of fungicide resistance‐associated mutations evolving in the closely related wheat yellow rust pathogen (Cook et al., 2021) is particularly concerning. It is vital that we continue to monitor and study outbreaks such as those in Ireland in 2020 to develop a better understanding of the inherent risk factors in this region that could support any future widespread epidemics.

## Supporting information


Figure S1

Figure S2

Figure S3

Figure S4

Table S1

Table S2

Table S3

## Data Availability

Sequence data from this article can be found in the European Nucleotide Archive (ENA) database at https://www.ebi.ac.uk under accession number PRJEB47693. Individual accession numbers for all publicly available sequence data used are provided in Table S1.
