# Supplementary figures and images for: Wheat stem rust recorded for the first time in decades in Ireland
Source: Plant Pathol. 2022 Feb 9;71(4):890–900. doi: 10.1111/ppa.13532 (PMC9303354; doi:10.1111/ppa.13532)

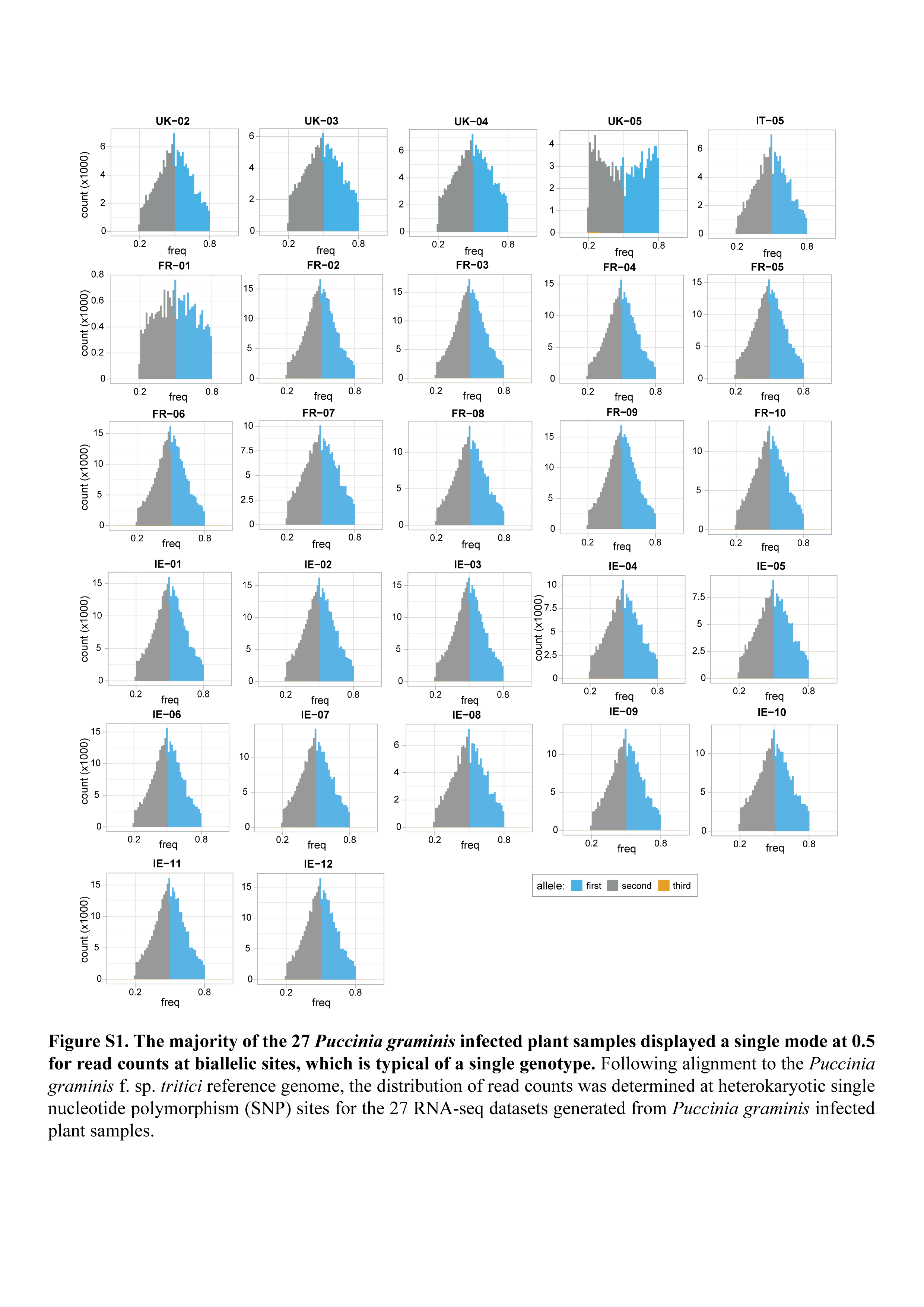

Supplement: Supplementary file 1 — Figure S1 [file PPA-71-890-s006.tif]

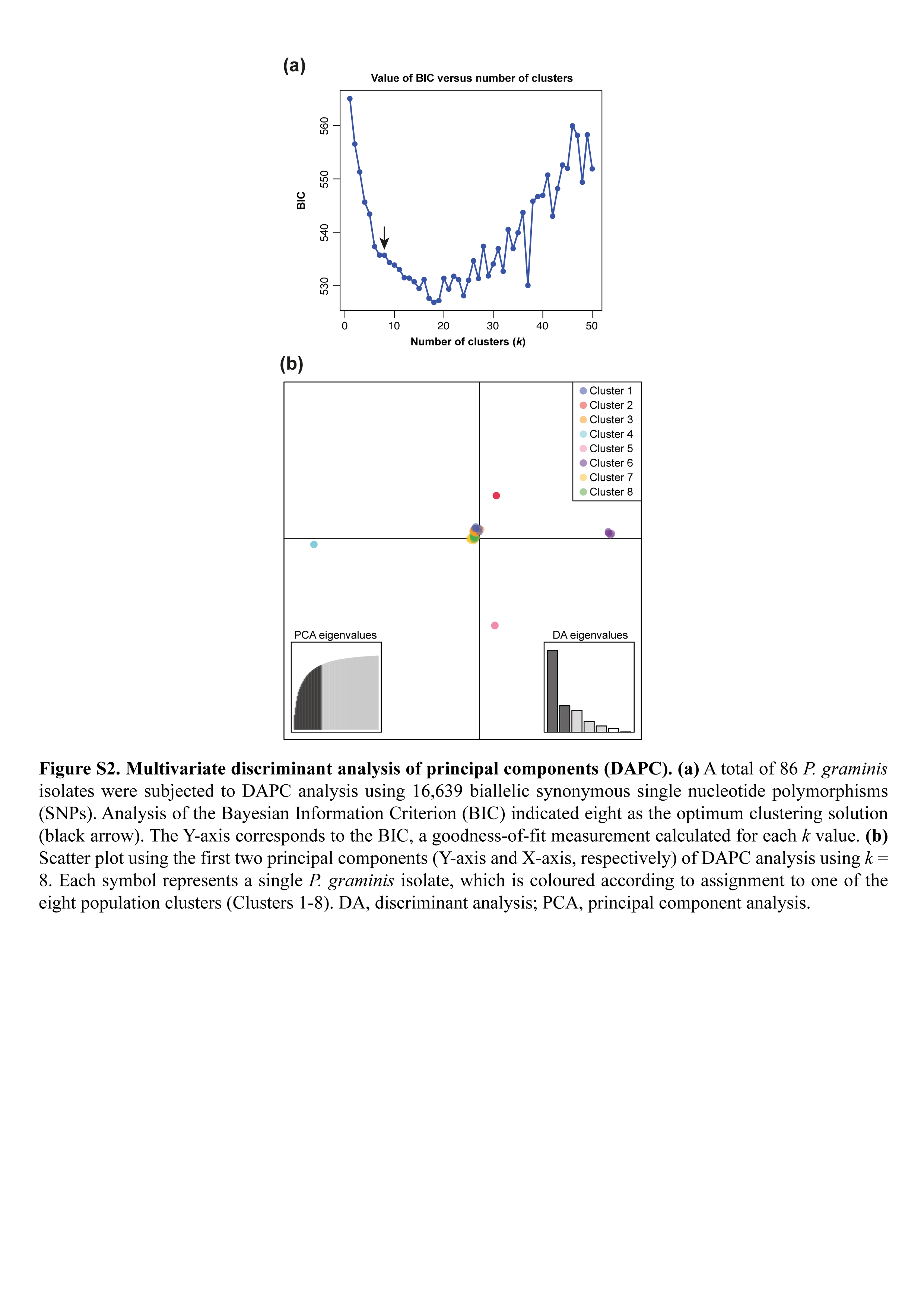

Supplement: Supplementary file 2 — Figure S2 [file PPA-71-890-s004.tif]

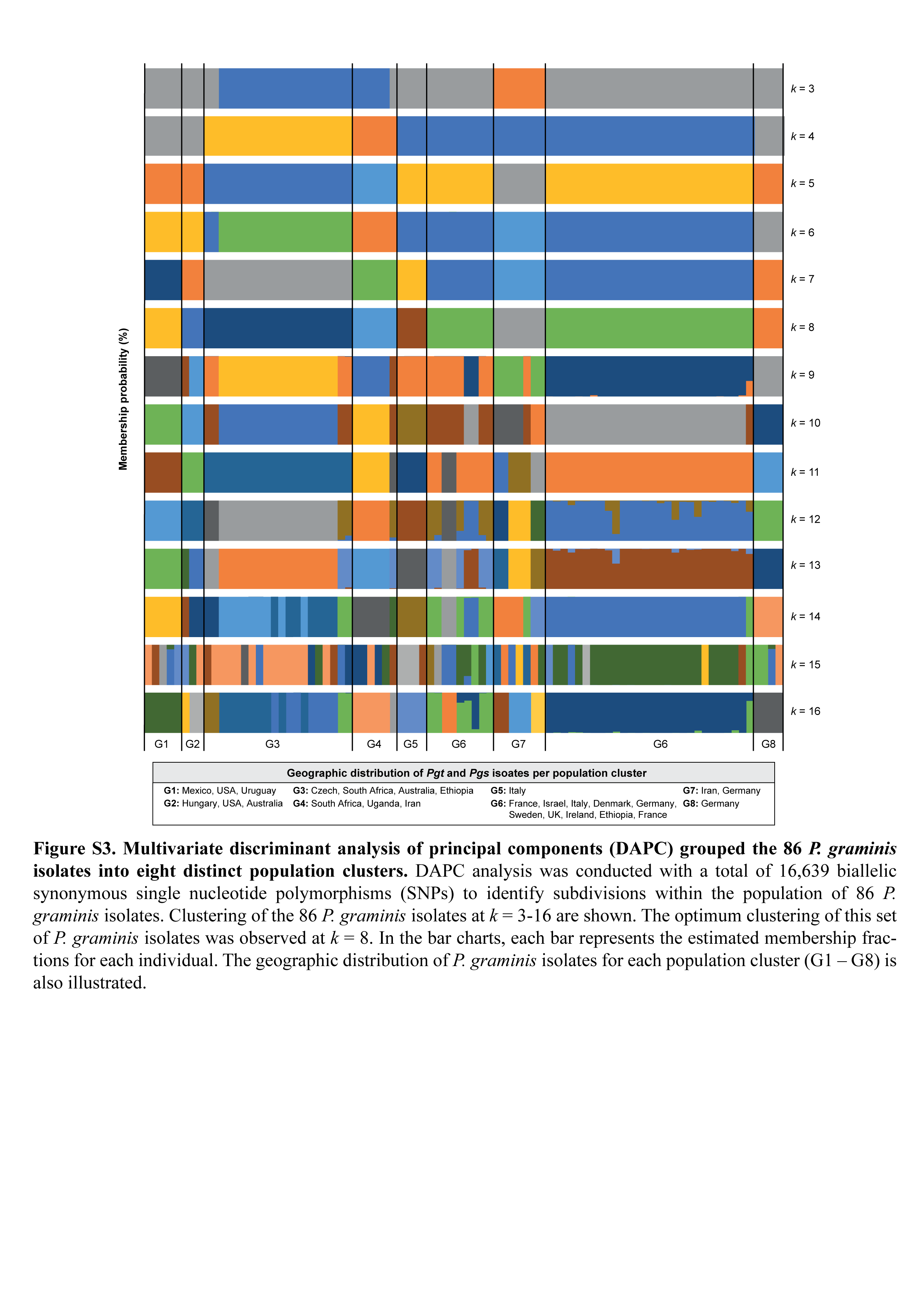

Supplement: Supplementary file 3 — Figure S3 [file PPA-71-890-s002.tif]

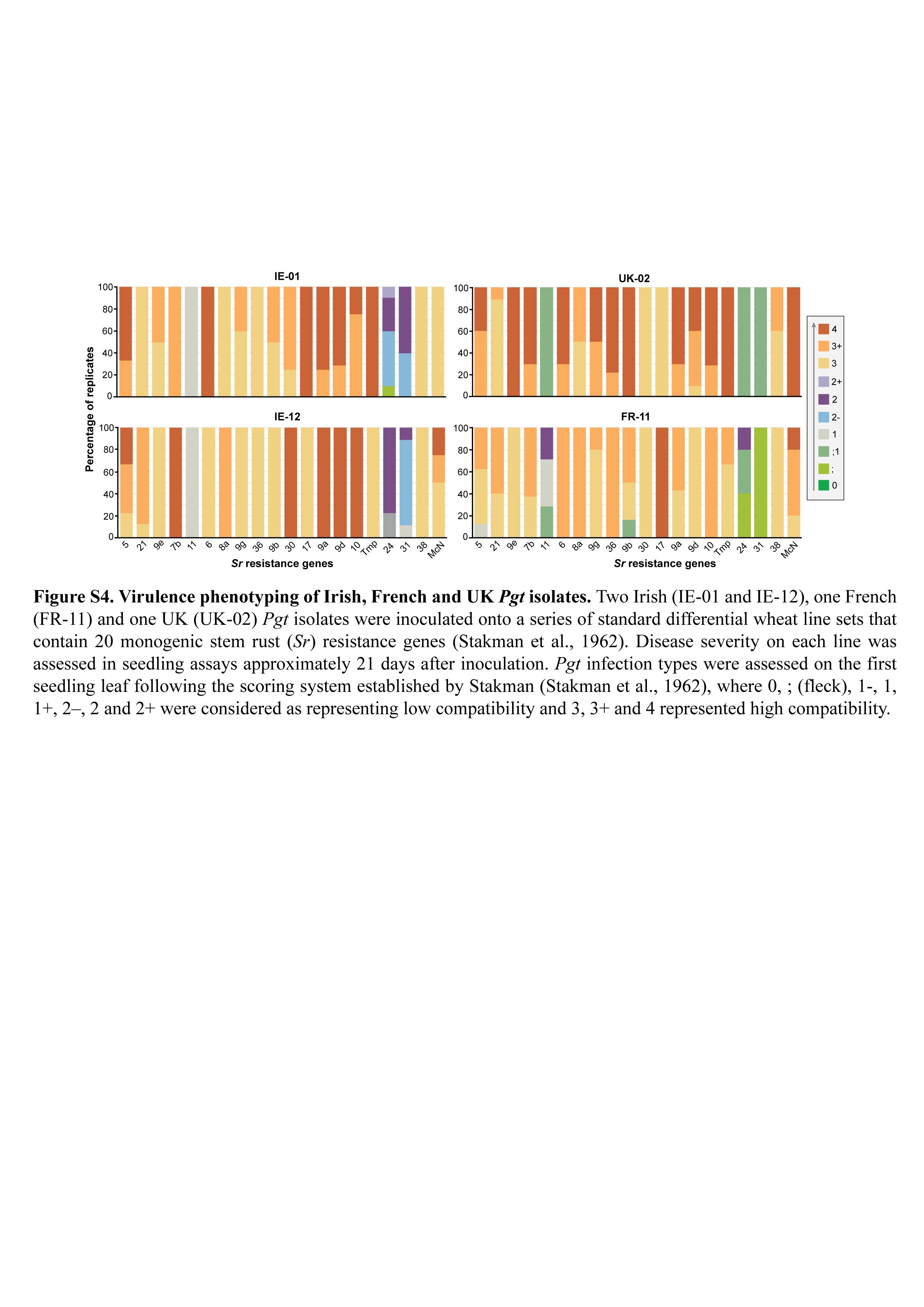

Supplement: Supplementary file 4 — Figure S4 [file PPA-71-890-s003.tif]
